# DOE-Assisted Formulation, Optimization, and Characterization of Tioconazole-Loaded Transferosomal Hydrogel for the Effective Treatment of Atopic Dermatitis: In Vitro and In Vivo Evaluation

**DOI:** 10.3390/gels9040303

**Published:** 2023-04-04

**Authors:** Rohini Kharwade, Nemat Ali, Purushottam Gangane, Kapil Pawar, Sachin More, Muzaffar Iqbal, Abid R. Bhat, Abdullah F. AlAsmari, Mohammed Kaleem

**Affiliations:** 1 Department of Pharmaceutics, Dadasaheb Balpande College of Pharmacy, Rashtrasant Tukadoji Maharaj Nagpur University, Nagpur 440037, India; 2 Department of Pharmacology & Toxicology, College of Pharmacy, King Saud University, Riyadh 11451, Saudi Arabia; 3 Department of Pharmacology, Dadasaheb Balpande College of Pharmacy, Rashtrasant Tukadoji Maharaj Nagpur University, Nagpur 440037, India; 4 Department of Pharmaceutical Chemistry, College of Pharmacy, King Saud University, Riyadh 11451, Saudi Arabia; 5 Department of Emergency Medicine, University of Maryland School of Medicine, 685 West Baltimore St, HSFI Rm 280I, Baltimore, MD 21201, USA

**Keywords:** tioconazole, transferosomes, drug release, histopathology, erythema score, in vitro

## Abstract

The present study was performed to determine the therapeutic effects of tioconazole (Tz)-loaded novel transferosome carriers (TFs) for the treatment of atopic dermatitis (AD). Method: Tioconazole transferosomes suspension (TTFs) was formulated and optimized using a 3^2^ factorial design. After that, the optimized batch of TTFs loaded into Carbopol 934 and sodium CMC was prepared with hydrogel and noted as TTFsH. Subsequently, it was evaluated for pH, spread ability, drug content, in vitro drug release, viscosity, in vivo scratching and erythema score, skin irritation, and histopathology study. Result: The optimized batch of TTFs (B4) showed the values of vesicle size, flux, and entrapment efficiency to be 171.40 ± 9.03 nm, 48.23 ± 0.42, and 93.89 ± 2.41, respectively. All batches of TTFsH showed sustained drug release for up to 24 h. The F2 optimized batch released Tz in an amount of 94.23 ± 0.98% with a flux of 47.23 ± 0.823 and followed the Higuchi kinetic model. The in vivo studies provided evidence that the F2 batch of TTFsH was able to treat atopic dermatitis (AD) by reducing the erythema and the scratching score compared to that of the marketed formulation (Candiderm cream, Glenmark). The histopathology study supported the result of the erythema and scratching score study with intact skin structure. It showed that a formulated low dose of TTFsH was safe and biocompatible to both the dermis and the epidermis layer of skin. Conclusion: Thus, a low dose of F2-TTFsH is a promising tool that effectively targeted the skin for the topical delivery of Tz to treat atopic dermatitis symptoms.

## 1. Introduction

Atopic dermatitis (AD) is one of the major skin diseases worldwide. It is a common and chronic inflammatory skin condition, in which the patient suffers intense itching with recurrent eczematous scratches [1]. It is associated with a number of health issues with an increased risk of allergic conditions, such as asthma and rhinitis, and is also associated with the opportunistic infection of Candida albicans. It also shows complex pathophysiological conditions, including a strong genetic tendency, epidermal dysfunction, and T-cell inflammation [2].Therefore, tioconazole (Tz), a broad spectrum antifungal compound, is used in the treatment of AD. It is applied against dermatophytes, different fungi concerned with deep mycosis, and some gram-positive and anaerobic bacteria. Due to conflicts in the systemic delivery of Tz, topical treatment for this infection is usually favored. Topical drug delivery dosage form directly targets the affected area, it results in improved patient compliance, decreased side effects, and sustained drug release. However, the stratum corneum is the main hurdle in the transdermal delivery system, because ionized and higher-molecular-weight molecules are unable to pass through this skin layer [3,4]. The poor skin penetration of the drug requires it to be delivered in a high dose during topical application, which causes side effects. Therefore, it is difficult to treat cutaneous disease through topical application [5,6]. Hence, to overcome the above-mentioned problems, it is essential to formulate and optimize a novel drug delivery system to treat AD by reducing the dose and side effects [4,7].

In recent years, transferosomes (TFs) have attracted more attention to overcome the penetration problem and act as potential carriers for topical drug delivery. TFs are ultra-flexible bilayer structured vesicles and offer sustained drug delivery for hydrophilic and hydrophobic drugs [8,9]. In addition to being penetration-enhancing mechanisms projected for liposomes, TFs can penetrate deeper skin layers because of their malleability, which is attributed to their ability to avoid dry environments [10,11].

Therefore, encapsulating the tioconazole (Tz) in transferosomes (TTFs) vesicles is one of the important approaches used to solve the problem of, and potentially treat, AD. The present work focused on the formulation of TTFs hydrogel (TTFsH). TTFsH ensured applicable drug localization within the skin and enhanced the therapeutic effects by improving penetration through the stratum corneum.

## 2. Results

### 2.1. Design of Tioconazole Transferosomes Suspension (TTFs)

Two different independent variables were used in the design and optimization of TTFs. They include amounts of phospholipon^®^90H (X_1_) and metallic element deoxycholate (X_2_), as shown in Table 1. Multilevel factorial design (3^2^) was used to screen the independent variables and nine different formulations of TTFs were obtained, as represented in Table 2. Further, formulations were evaluated for particle size, PDI, and entrapment efficiency to determine the optimized formula. The response surface regression analysis produced a mathematical relationship between the components and the parameters.

**Figure 1:**
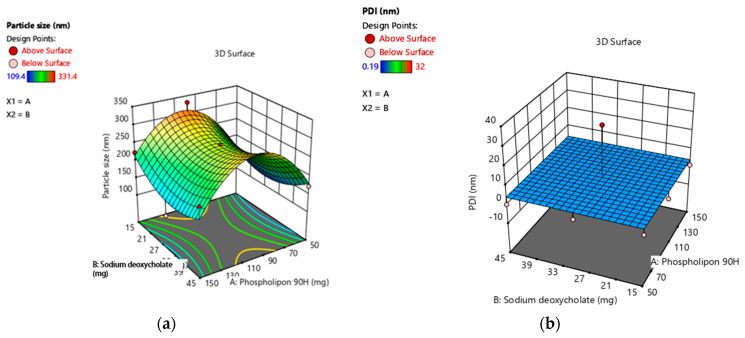

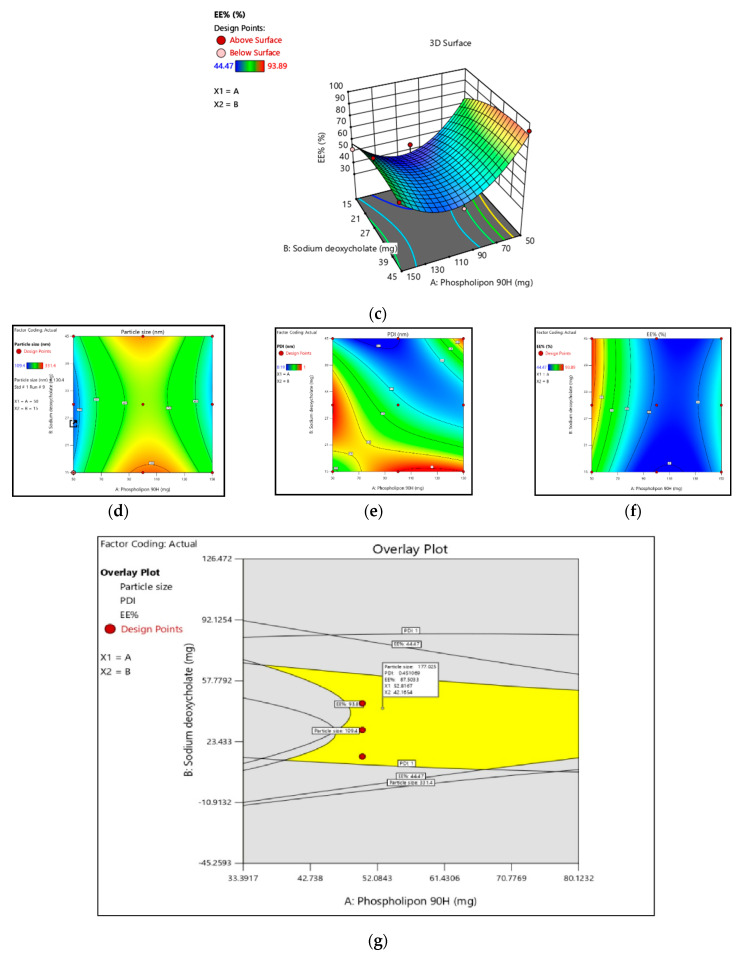
(**a**–**c**): Three-dimension surface response plot showing the effect of phospholipon^®^90H concentration with respect to sodium deoxycholate concentration over the particle size, PDI, and % EE, respectively. (**d**–**f**): Contour plot showing the effect of Phospholipon^®^90H concentration with respect to sodium deoxycholate concentration over the particle size, PDI, and % EE, respectively. (**g**): Overlay plot of optimized area for batch B4 (in yellow). Legend of Figures in Appendix A.

Using design-expert software, all of the observed responses for the nine produced formulations were fitted to various models. The best models were found to be quadratic for particle size, PDI, and %EE. Figure 1 illustrates the effect of X_1_ and X_2_ on the particle size (Y_1_), PDI (Y_2_), and EE% (Y_3_) of TTFs [12,13].

The contour plot and the estimated response surface showed that the independent variables X_2_, decreased particle size; however, X_1_ increased the particle size (Y_1_), as shown in Figure 1a,d. The vesicle size fluctuated from 109.4 ± 0.604 nm to 331.4 ± 0.684 nm, which confirmed that all prepared TTFs have a vesicle size in the nanometer range. Therefore, it was effective for transdermal applications. Equation (1) indicated the quadratic interactive models for the vesicle size of TTFs, as obtained from a factorial design study.

Y_1_ = 178.35 + 16.83 X_1_ − 14.43 X_2_ + 2.33 X_1_ X_2_ + 42.43 X_1_^2^ + 12.33 X_2_^2^ (1)

As shown in Table 3, the F-value was 21.09, and the adjusted and predicted R^2^ values were about one. Therefore, It was confirmed that the model was best suited, and terms X_1_, X_1_^2,^ and X_2_^2^ showed a significant effect on TFs size.

Particle homogeneity is indicated by the PDI, and it varies from 0.0 to 1.0, indicating the size distribution of a sample. All formulations showed narrow and homogenous size distribution as represented in Table 4. This indicates higher physical stability and more uniform vesicle size. The quadratic interactive model for the PDI of TTFs was represented in Equation (2), as obtained from a multilevel factorial design study.

Y_2_ = 93.43 + 3.24 X_1_ + 0.34 X_2_ − 0.17 X^1^ X^2^ + 0.19 X_1_^2^ + 0.32 X_2_^2^ (2)

As shown in Table 3, the F-value was 26.03, and the adjusted R^2^ and predicted R^2^ values were about 1 for the above model Equation (2). This confirmed that the model was significant and that terms X_1_, X_1,_ X_1_^2^, and X_2_^2^ have a significant effect on PDI and its effects, as shown in Figure 1b,e.

It was observed that independent variables X_1_ and X_2_ both showed a positive effect in % drug entrapment efficiency (EE%) with values ranging from 44.86 to 93.89. As shown in Figure 1c,f, the contour plot and the estimated response surface variables X_1_ and X_2_ improved the entrapment efficiency. The quadratic interactive model for the EE% of TTFs was represented in Equation (3). It was found that the EE% was significantly increased with additional increases in the concentration of sodium deoxycholate, as shown in Table 5. However, it decreased with an increase in the concentration of phospholipon^®^90H. This happened because the lipid bilayer structure of phospholipon^®^90H compressed the vesicle and, therefore, decreased the %EE [14].

Y_3_ = 93.43 + 3.24 X_1_ + 0.34 X_2_ − 3.04 X_1_ X_2_ − 24.43 X_1_^2^ − 0.72 X_2_^2^ (3)

As shown in Table 3, the F-value was 23.07, and the adjusted and predicted R^2^ values were about 1 for the above model Equation (3). Therefore, it confirmed that X_1_ and X_2_ both showed the imperative effect on the entrapment efficiency of TTFs.

### 2.2. Optimization of Formulation Variable

Per the result of these quadratic models, the optimum ratio of X_1_ and X_2_ was selected to prepare TTFs, which showed a potential effect in the treatment of AD. In the response surface, the overlay plot showed the optimized area for batch B4 in yellow, as shown in Figure 1g, and the formulation of TTFs, as shown in Table 5.

**Figure 2:**
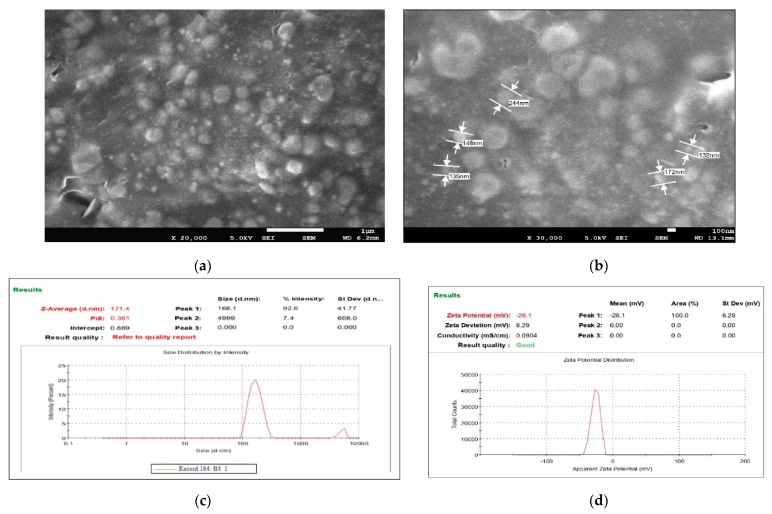
FE-SEM images of B4 optimized batch of Tz-loaded transferosome suspension (TTFs) (**a**,**b**), which was spherical, well identified, unilamellar nanovesicles Vesicle size and zeta potential of the optimum batch of TTFs. Batch B4 had a vesicle size, of 171.4 nm ± 9.03 with PDI 0.36 (**c**) zeta potential 26.13 (**d**) which facilitated to cross of the stratum corneum and stability to the TFs vesicles.

From the characterization of all nine batches, it was observed that all predicted parameters of batch B4 were closer to the predicted values of the software. It showed a vesicle size, PDI, and a % drug entrapment value of 171.4 nm ± 9.03, 0.36 ± 0.04, and 93.89% ± 2.41, respectively, as shown in Table 4. Optimized TTFs appeared as spherical, well identified, unilamellar nanovesicles, as shown in SEM photographs of Figure 2a,b. All batches have a negative zeta potential value ranging from −0.873 mV to −26.13 mV, as shown in Table 4. The values of zeta potential also showed a negative charge after Tz was loaded into TFs. It suggested that the anionic charge of the drug and sodium deoxycholate did not dominate over the lipids. The zeta potential of the B4 batch was 20.73 ± 1.23 mV, as shown in Figure 2c,d.

### 2.3. Drug and Excipients Interaction Study

FTIR spectrophotometry has been used to identify the drug–excipient interaction.

**Figure 3:**
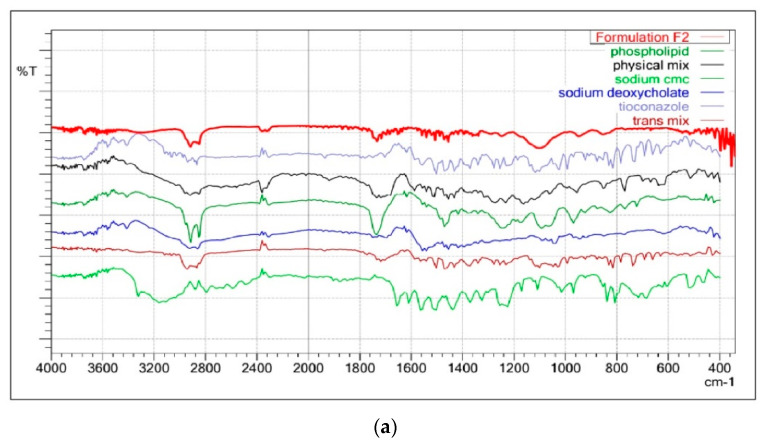

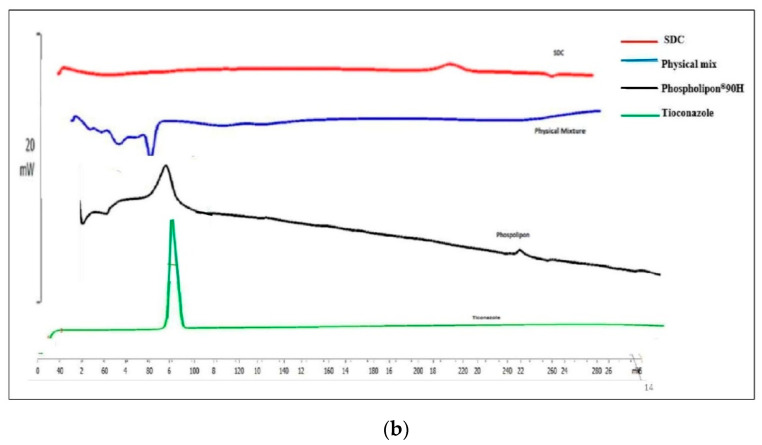
(**a**): FTIR overlay of Tz, TTFs, and TTFsH indicated the coordination of Tz to phospholipon^®^90H and a sodium deoxycholate complex. (**b**): DSC thermogram of Tz and TTFs predicted the amorphous or partially amorphous state of Tz in TTFs, which led to a high-energy state and high disorder, resulting in enhanced solubility.

Figure 3a illustrated the FTIR spectrum of Tz, TTFs, and TTFsH (F2). The FTIR spectra of Tz were characterized by bands at 3181 cm^−1^ for N-H and 723.31 cm^−1^ for -C-S stretching, and a sharp peak at 1255.66 cm^−1^ for -C-N, 1328 cm^−1^ for -C-N stretching, and 1433.11 cm^−1^ for -CH_2_ bending. In the TTFs, the absence of a vibration peak related to the N-H group of amines and the appearance of a new strong peak at 1730 cm^−1^ have suggested the formation of a new imine complex of phospholipon^®^90H and of sodium deoxycholate with Tz. The other peaks of Tz were similar in TTFs and TTFsH; however, there was a distinct change in the peaks at 1232.51 cm^−1^ for imidazole -C-N stretching, 1433.11 cm^−1^ for -CH_2_ bending, and 769.80 cm^−1^ for thiol -C-S stretching. These slight changes in the position of stretching frequencies of Tz in the TTFs and TTFsH formulations concluded the coordination of Tz to phospholipon^®^90H and the sodium deoxycholate complex.

The solubility and physical state of Tz in lipid nanovesicles were characterized by DSC. The DSC analysis of the pure Tz and the physical mixture of TTFs with phospholipon^®^90H and sodium deoxycholate were studied. The DSC analysis for pure Tz showed a sharp crystalline peak at 84.89 °C, as shown in Figure 3b, which represents the crystallinity of the drug. However, this peak disappeared in the DSC thermogram of TTFs.

### 2.4. In Vitro Permeation Study of Tioconazole Transferosomes (TTFs)

**Figure 4:**
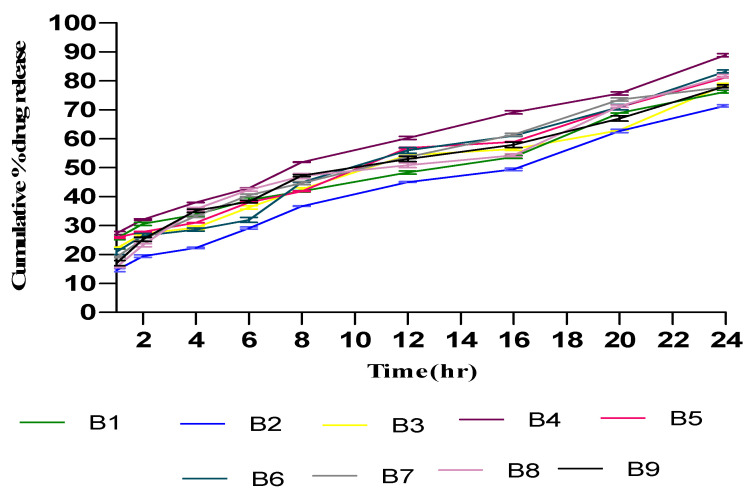
In vitro permeation studies of Tz-loaded TFs in saline phosphate buffer of pH 6.5 using Franz diffusion cell with freshly shredded dorsal rat skin (±SD, n = 3). B1, B2, B3, B4, B5, B6, B7, B8, and B9 are the tioconazole transferosome batches.

An in vitro permeation study of TTFs was accomplished in a phosphate buffer of pH 6.5 using Franz diffusion cells with freshly shredded dorsal rat skin. The in vitro permeation profile of Tz is represented in Figure 4, which shows the biphasic release of Tz. At the initial phase, Tz permeated rapidly, which persisted for about 8 h followed by a slow permeation phase that continue for 24 h. The transdermal flux of all prepared TTF batches had noticeably higher flux values with a range from 31.12 ± 1.28 µg/cm^2^/h to 48.7 ± 1.08 µg/cm^2^/h. The permeability results of the optimized batch (B4) observed 89% cumulative Tz release for the 24 h with a flux of 48.23 ± 0.42. Similarly, the Tz concentration for B1, B2, B3, B4, B5, B6, B7, B8, and B9 present in skin tissue were found to be 7.23 ± 0.31, 5.12 ± 0.42, 6.87 ± 0.15, 10.04 ± 0.39, 8.17 ± 0.17, 8.02 ± 0.30, 6.98 ± 0.32, 8.98 ± 0.47, and 7.76 ± 0.21 µg/cm^2^ (mean ± SD, n = 3), respectively, after 24 h. It also showed that the B4 batch of TTFs retained more Tz in skin tissue as compared to other batches.

### 2.5. Characterization and Optimization of Tioconazole Transferosomal Hydrogel (TTFsH)

The optimized batch (B4) of TTFs was loaded into different batches of hydrogel base, according to different concentrations of Carbopol 980 and sodium CMC. Subsequently, it was evaluated for pH, viscosity, spreadability, and drug content, as well as in vitro drug release.

The optimized TTFsH (F2) was prepared using 1.5% sodium CMC as a gelling agent. All batches with an equivalent amount of 2% *w/w* of Tz containing TTFsH were smooth and homogeneous in appearance. The spreadability and viscosity of the optimized F2 batch were found to be 28.6 ± 0.24 cm and 4.94 Pa/s, respectively, which indicated that it can be spread easily on the skin surface with little stress. The viscosity and spreadability were slightly greater in Carbopol 980-based hydrogel compared to sodium CMC-formulated hydrogel. The greater swelling index value of Carbopol 980, compared to sodium CMC, was responsible for the viscosity and spreadability of the hydrogel.

The pH was found to be in the neutral range from 6.1 ± 0.16 to 7.1 ± 0.23, which is essential for topical preparations. The % drug content in the optimized TTFsH batch was found to be 95.72% ± 2.90, as shown in Table 6, which represented good content uniformity.

### 2.6. In Vitro Drug Release Study

**Figure 5:**
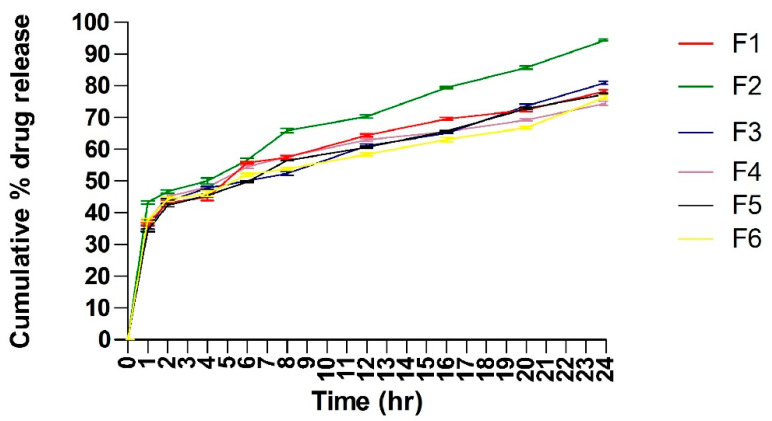
Cumulative % Tz release from different batches of TTFsH. F2 batch showed the highest % Tz release by following a Higuchi drug release mechanism. It showed an initial burst release followed by slow and sustained release of Tz (±SD, n = 3). F1, F2, F3, F4, F5, and F6 are transferosome-loaded hydrogel batches.

The release profiles of Tz from different batches of prepared TTFsH were illustrated in Figure 5. It was found that the cumulative % drug release from all batches was greater. Due to the high flexibility of TFs, they can easily penetrate the skin by overcoming the intracellular lipid barrier of the stratum corneum [9,15]. This drug release data and the drug release mechanism were used to optimize the TTFsH batch. The optimized (F2) batch released 94.23 ± 0.984%, with a flux of 47.23 ± 0.82, which was significantly higher compared to the other batches of TTFsH. The F2 batch easily spread on the stratum corneum layer and diffused to a more hydrated dermis and the epidermis layer due to the effect of an osmotic gradient. The role of Tween 80 in the structure of the TFs was to support the solubilization of lipids in the stratum corneum and enhance drug penetration. Additionally, it provided a large surface area for the dissolution of a drug in the hydrogel base and maintained a concentration gradient for diffusion [16].

Different kinetic models were applied to the results of the in vitro release studies and were used to calculate the regression coefficients (R^2^), as shown in Table 7. The Tz released from all investigated TTFsH batches followed a Higuchi model with a correlation coefficient greater than zero and first-order kinetics. The release rate of Tz showed F2 > F5 > F3 > F1 > F4 > F6, which differed depending on the type and concentration of the gelling agent used in the hydrogel. The Korsmeyer–Peppas release exponent data showed that the F2 batch of TTFsH was described as a non-Fickian anomalous release with an n value of 0.533 (which is <0.5) [17].

### 2.7. In Vivo Study of Scratching Score and Erythema Score

A DNCB-induced AD model of an SD rat was created to consider erythema, edema, and scabs, and scratching behavior. Subsequently, rats in groups III, IV, V, and VI were treated with hydrogel base, a market preparation, TTFsH (low dose), and TTFsH (high dose), respectively. The scratching score and erythema score were monitored and quantified by every single rubbing of their dorsal skin layer and ears with their hind paws.

**Figure 6:**
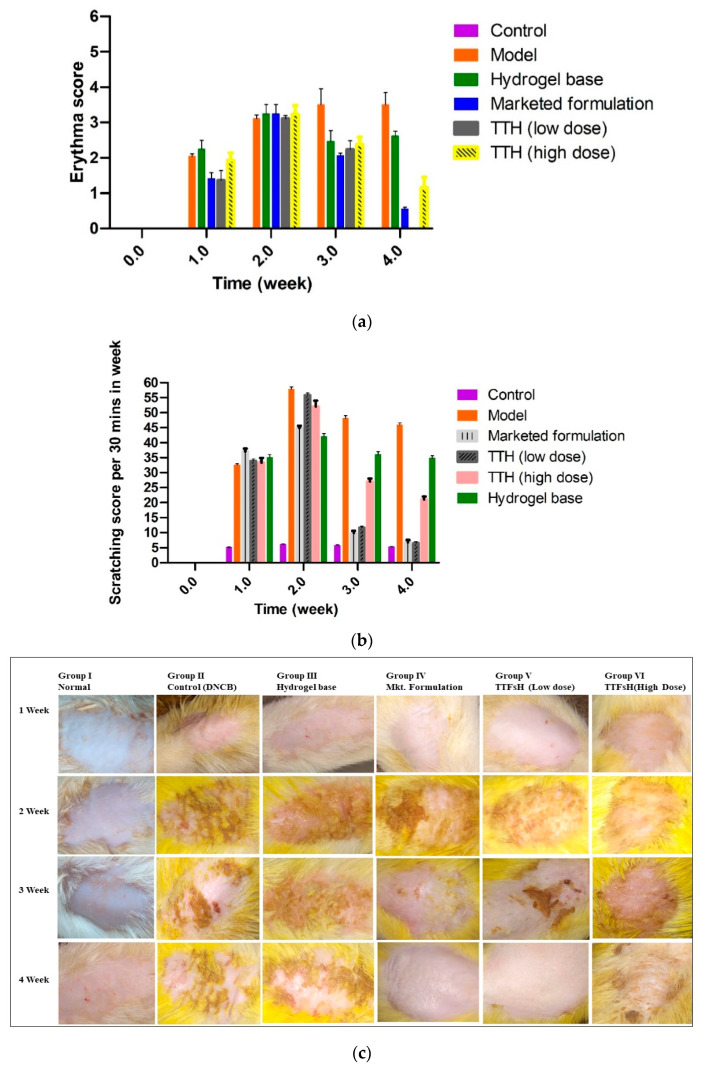
Four weeks of treatment with TTFsH (low dose) resulted in significant recovery of the skin compared to the marketed formulation, as shown from decreased scratching score (**a**) and erythema score (**b**) (±SD, n = 3, *p* < 0.5). Clinical features of SD rat skin after the topical treatment of hydrogel base, marketed formulation (Mkt. formulation), TTFsH (low dose), and TTFsH (high dose) on DNCB-induced AD (**c**).

After administration of TTFsH (low dose) for 4 weeks, the injured skin started to recover and the wound produced a scab. In this process, the scabs progressed to healing skin with new hairs growing on the damaged stratum corneum. The clinical features of the SD rat’s skin also improved, as shown in Figure 6c. The model and plain hydrogel-based group showed scratching scores of 45.01 ± 1.04 and 35.01 ± 0.86, respectively. The scratching score was significantly decreased following a low dose of TTFsH (from 56.43 ± 1.34 to 6.57 ± 0.92) on the fourth week of the experiment in DNCB-induced AD rats, as shown in Figure 6a. However, the scratching score increased following high doses of TTFsH. Thus, this proves that the low dose of TTFsH retained Tz in different skin layers, and took effect quickly, compared to the high dose. Low-dose TTFsH treatment can lower scratching and erythema scores to the same degree as market products, as shown in Figure 6b.

### 2.8. Histopathological Examination

**Figure 7:**
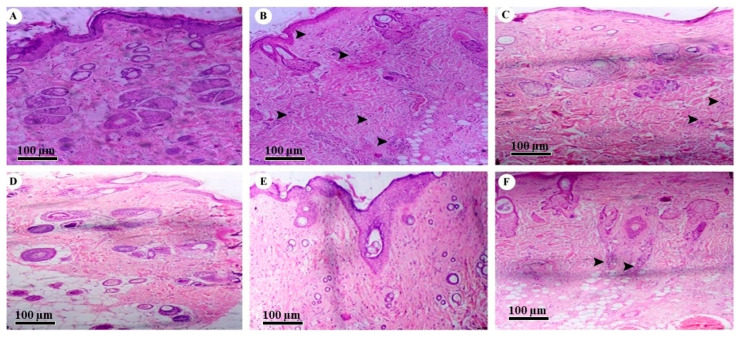
Histopathology of (**A**) normal skin tissue, (**B**) model group (induced by DNCB), (**C**) tissue treated with hydrogel base, (**D**) tissue treated with marketed formulation, (**E**) tissue treated with TTFsH (low dose), and (**F**) tissue treated with TTFsH (high dose).

The histopathological section of normal SD rat skin showed uniform dermis and epidermis with a healthy capillary loop, as shown in Figure 7A. Arrows were added to Figure 7 to highlight inflammatory cell infiltration and leukocytes, and the figure used the bar scale of 100 µm. According to the literature, AD is believed to arise when the immune system of the body triggers abnormal or overactive inflammatory responses in the dermis and epidermis [18]. All of these changes were observed in the histopathological skin section of the positive control group, which was treated with DNCB. The group showed focal hemorrhage along with inflammatory cell infiltration and leukocytes in the dermis and muscle with a mild compact hyperkeratosis layer, as shown in Figure 7B. Focal interface dermatitis was also seen in the positive control group with chronic inflammation and a dense dermis layer. However, the hydrogel base-treated group showed normal skin structure, as shown in Figure 7C. While the market formulation-treated group showed recovered dermis and epidermis cell structure, there was no change observed in focal acanthosis or skin appendages, and there was an absence of leukocytes, as shown in Figure 7D. Simultaneously, the histopathological section showed that the skin layer became significantly thinner and the number of inflammatory cell nuclei also declined, as shown in Figure 7D. Similarly, in Figure 6c also showed the significant increased hair growth and improved skin healing.

The SD rats that received the low dose of TTFsH showed an even and unbroken dermis and epidermal layer. The group also observed a thinner epidermis and reduced inflammatory cell infiltration in subcutaneous adipose tissue, as shown in Figure 7E. However, rats treated with a high dose of TTFsH had slight inflammatory cell infiltration and leukocytes in subcutaneous adipose tissue. A foreign body granuloma was also seen in the dermis layer of the skin (Figure 7F). The granuloma was formed by the aggregation of histiocytes and multinucleated giant cells with hair burrows in the nucleus [19]. The observed results of histopathology supported the results of erythema and scratching scores represented in Table 7 and Figure 7.

## 3. Discussion

In the present investigation, tioconazole (Tz) was encapsulated in transferosome (TTFs) vesicles to ensure drug localization within the skin and enhance the therapeutic efficacy of AD treatment. The TFs were formulated and optimized by a multilevel factorial design (3^2^) using two independent variables, phospholipon^®^90H (X_1_) and sodium deoxycholate (X_2_), and nine formulated batches. From the observations, it was found that the quadratic model was best fitted for designing spherical nanovesicles and uniform distribution with the highest entrapment efficiency. Sodium deoxycholate decreases the surface free energy, ultimately increasing total surface area and decreasing particle size. Therefore, the particle size decreased as sodium deoxycholate increased [20]. It was also found that the particle size was reduced with an increased sodium deoxycholate–surfactant ratio. A minimum quantity of surfactant ensured incomplete maturation of vesicles and, thus, reduced their size [21]. Therefore, the optimum concentration of phospholipon^®^90H and sodium deoxycholate with Tween 80 showed vesicle size in nanoscale range. It also showed the best results for deformability and % EE. The TFs have a high degree of deformability compared to other lipid vesicles. All batches showed negative zeta potential, which prevented the coagulation of vesicles, and their magnitudes provided stability to the TFs [22,23]. Each parameter for the B4 batch was optimized, with vesicle size, PDI, % drug entrapment, and zeta potential values of 171.4 nm ± 9.03, 0.36 ± 0.04, 93.89% ± 2.41, and −20.73 ± 1.23, respectively.

The formulation of this phospholipid complex with Tz was ascertained by FTIR and DSC. In the FTIR, the appearance of a new strong peak at 1730 cm^−1^ has suggested the formation of a new imine group in place of amine, which suggests the coordination of Tz to a phospholipid complex. DSC analysis suggested that the Tz was transformed into a more soluble amorphous state when loaded into TFs. This high-energy amorphous or partially amorphous state of Tz increased its solubility under TFs [24,25].

The in vitro permeation study showed the capacity of transferosomes to penetrate from the drug into the skin. A result of this study was that the transdermal flux of all prepared TTF batches was high and showed a biphasic release profile. The initial rapid release was observed due to the adsorption of Tz, and the slower release was observed because of the entrapment of Tz in the lipid vesicles. The lipid bilayer of TFs spontaneously deformed without breaking the structure of the lipid when stress was applied to vesicles and the drug easily penetrated the skin pores [26]. Therefore, TFs increased the retention levels of Tz in the skin, and might be a promising molecule for the therapy of AD. The nanosized ultratransformable nature of the TFs might increase the interfacial area, which affects drug transportation and contributes to improving the absorption of Tz from TTFs [27,28].

Furthermore, the optimized batch of TTFs (B4) with 2% *w/w* of Tz was homogeneously mixed with different concentrations of Carbopol 980 and sodium CMC hydrogel base and evaluated for optimization. From the release profile and drug content, the F2 batch of TTFsH, which contained 1.5% sodium CMC as a gelling agent, was optimized. The viscoelastic moduli, molecular weight, and density of the sodium CMC are responsible for arranging themselves in a network form [29,30]. Thus, the drug content and release profile of sodium CMC containing hydrogel were better. The in vitro drug release profile of TTFsH showed the process in which Tz migrates from the polymeric system to the outer surface of the polymeric system, which leads to the release into the medium. The study of the release followed a Higuchi model, which indicated that the amount of Tz released from TTFsH was the square root of the time-dependent process and the diffusion mechanism. This mechanism showed an initial burst released during the 4 h when the concentration of the dissolved drug in the hydrogel vehicle was high. It was followed by a sustained release for the duration up to 24 h. This happened because Tz was entrapped in TFs and the fraction of the Tz particles were dispersed in the homogeneous hydrogel matrix [31,32]. The Korsmeyer–Peppas release exponent data showed a non-Fickian anomalous release with an n value of 0.533. This release occurs by the usual diffusion and matrix erosion. Therefore, it could be concluded that the continuous release of Tz was dependent on the partitioning of Tz in TF phospholipid vesicles and its diffusion from the gel network structure [33,34].

However, the DNCB-induced AD model of an SD rat showed prominent thickening of the stratum corneum, with the aggregation of the inflammatory cell nucleus, as shown in Figure 6b, confirmed in the AD model. After the treatment of TTFsH, the scratching score was significantly decreased by the treatment of TTFsH (low dose). The histopathological section of the skin also supported the results of the scratching and erythema score study. It also showed intact infiltration of inflammatory cells in the dermis to the same extent as the marketed formulation with a low dose with a reduced dose-dependent side effect.

Hence, TFs generated the osmotic gradient due to the difference in water content between the stratum corneum and the epidermis layer. It helps to transfer lipids across the skin and promotes penetration of the drug by the disruption of the highly ordered lipid structure, which allowed greater penetration and deposition of Tz in the deeper skin layer to treat AD [35,36].

## 4. Material

### 4.1. Chemicals

Tioconazole was procured as a gratis sample from Enaltec Lab. Pvt. Ltd. (Mumbai, India). Phospholipon^®^90H was a gift sample from Lipoid GmbH, (Ludwigshafen am Rhein; Germany), and Carbopol 980 was a gift sample from Lubrizol Advanced Materials Republic of India Pvt. Ltd., Mumbai, India. Sodium deoxycholate was a gift sample from Himedia Lab. Mumbai. All the chemicals used for experimental purposes were of an analytical grade and were purchased from Sigma Aldrich; (St. Louis, MO, USA).

### 4.2. Animals

Male Sprague Dawley (SD) rats were sanctioned by the institutional animal ethical committee (IAEC) as per CPCSFA guidelines for scratching and erythema scores, the irritation study, and histopathological studies with approval numbers DBCOP/IAEC/1426/21-22/P2. Rats were 200 ± 50 g and kept in an environmentally controlled breeding room at the temperature of 22 ± 2 °C with a humidity of 50% RH. All animals had access to sufficient food and water.

### 4.3. Design of Experiments and Statistical Data Analysis (DOE Version 13)

TFs were developed by optimizing the phospholipon^®^90H and metallic element deoxycholate quantitative relations of two prime selected independent variables. For this, 3^2^ factorial styles with 2 variables and phospholipon^®^90H (X_1_) and sodium deoxycholate (X_2_) at 3 levels (low, medium, and high) were applied, as shown in Table 1 and Table 2 [22,37]. These two factors were varied at three levels, vesicle size (Y_1_), PDI (Y_2_), and drug entrapment efficiency (EE%) (Y_3_), regarding drug-loaded transferosome suspensions. These responses were glorious to play a very important role in determining the dose regime of an entrapped drug [12,38]. DOE version 13 software (Design knowledgeablethirteen packages from Stat- Ease Inc., Minneapolis, MN, USA) was used for experimental design and statistical data analysis.

### 4.4. Formulation of Transferosome Suspension (TTFs)

A thin lipid film hydration method was used for the formulation of TFs with some modifications [39,40]. In a clean round-bottomed, flask different concentrations of phospholipids, wetting agent (edge activator), and 500 mg of Tz were solubilized in an organic solvent containing chloroform and methyl alcohol (2:1 *v/v*). Then, it was rested at room temperature for 24 h until the formation of a thin film on the inner surface of the round- bottomed flask. The mixture of the organic solvent was then evaporated using a rotary evaporator at a temperature of no more than 45 °C under low pressure. The solvent residue was permitted to disappear with an overnight vacuum. Subsequently, a solution of sodium deoxycholate (edge deactivator) in saline phosphate buffer (pH 7.4) was added and it was stirred at 60 rpm for 1 h at room temperature to hydrate the film. It had been allowed to swell for 2 h and further sonicated for 15 min to obtain TFs [41,42].

### 4.5. Characterization of Tioconazole-Loaded Transferosomes Suspension (TTFs)

#### Drug and Excipients Interaction Study

The TTFs were analyzed for drug excipient interaction study by FTIR and DSC [43,44]. Further, all batches were also analyzed for %EE [43,45]. The particle size, polydispersity index (PDI), and zeta potential of TTFs were determined using the dynamic light scattering technique at 25 °C [43,46]. FE-SEM was also used to confirm the particle size, shape, pores, and aggregation along with the morphology of TFs [42].

### 4.6. In Vitro Permeation Studies of TFs

A Franz diffusion cell apparatus with an effective surface area of 2.50 cm^2^ was used for in vitro permeation study of TTFs. The study was performed with depilatory agents and freshly shredded dorsal rat skin, which was moisturized using a regular saline solution. This prepared rat skin was fitted in between the donor and receptor compartments of the diffusion cell. The stratum corneum layer faced toward the donor compartment and the dermis layer faced the receptor compartment, which contained 30 mL ethanolic saline phosphate buffer with a pH of 6.5 (1:1 *v/v*). The temperature of the receptor compartment was kept constant at 37 °C ± 0.5 °C for 24 h and stirred at 50 rpm [47]. The TTFs (equivalent to 50 mg of Tz) were uniformly spread on the stratum corneum. At different time intervals (1, 2, 4, 6, 8, 10, 12, and 24 h) 2 mL sample was withdrawn from the receptor compartment and an equal volume of fresh dissolution medium was added to it for the maintenance of sink condition. The permeated concentration of Tz was analyzed by UV-spectrophotometer at 224 nm against ethanolic phosphate buffer with a pH of 6.5 (1:1 *v/v*). The cumulative % of Tz released was plotted against time and the value of steady-state flux was determined [48].

The skin surface was cleaned 5 times with ethanolic phosphate buffer with a pH of 6.5 (1:1 *v/v*), and then cleaned with water to eliminate extra TTFs from the surface after 24 h. The skin tissue was homogenized with a dissolution medium and rested at room temperature for 6 h. It was diluted with ethanolic phosphate buffer with a pH of 6.5 (1:1 *v/v*) and centrifuged at 5000 rpm for 15 min. The supernatant liquid was assessed for the Tz concentration present in skin tissue using a UV-visible spectrophotometric technique at 224 nm [49,50].

### 4.7. Formulation of TTF-Loaded Hydrogel (TTFsH)

The 2% *w/w* of Tz-containing hydrogel was prepared with different percentages of Carbopol 980 and sodium carboxymethyl cellulose (sodium CMC) as a gelling agent. Methylparaben, propylparaben, and sodium metabisulphite as a preservative were dissolved in 50 mL of water. The accurate amount of Carbopol 980 and sodium CMC was added according to different batches, as shown in Table 8, and kept at room temperature overnight. An amount of 1 mL triethanolamine as a crosslinking agent was slowly added into the above gel base with continuous stirring. Subsequently, the optimized TTFs containing an equivalent of 2% *w/w* Tz was added into it with continuous stirring to form a homogeneous gel. At last, the pH of the gel was adjusted to pH 7 by dropwise addition of sodium hydroxide with continuous stirring [51,52].

### 4.8. Characterization of TTFsH

All prepared batches of TTFsH were evaluated for pH, homogeneity, viscosity, spreadability, drug content, in vitro drug release, and in vivo erythema score and scratching score, and in a histopathology study [53,54].

#### 4.8.1. In Vitro Drug Release Study

The in vitro drug release of Tz from TTFsH was studied by using a Franz diffusion cell at 37 ± 0.5 °C. A cellulose dialysis membrane with a molecular weight of 12,000 Da, a pore size of 2.4 nm (Hi-Media, Mumbai, India), and an effective surface area of 2.50 cm^2^ was used. The phosphate buffer with a pH of 7.4 was used as a dissolution medium in the receptor compartment. The experimental procedure was the same as that of the in vitro drug permeability study of TTFs [55,56].

The % drug release was kinetically analyzed by zero-order, first-order, Higuchi model, and Korsmeyer–Peppas model to determine the drug release mechanism. The release exponent of Tz was studied by Korsmeyer–Peppas Equation (4).

Q_t_/Q_o_ = K_P_t^n^ (4)

where Q_t_ is the amount of Tz released in time t; Q_o_ is the initial amount of Tz in the transferosomes; Kp is the Korsmeyer–Peppas release rate constant; n is the release exponent indicative of the mechanism of release from spherical matrices. Note: A Fickian diffusion-mediated drug release occurs if 0.43 < n < 0.5, while a non-Fickian diffusion-mediated drug release is predominant if 0.5 < n < 1 [57,58,59].

#### 4.8.2. In Vivo Study of Scratching Score and Erythema Score

In this study, in vivo erythema and scratching scores of AD-induced SD rats were determined. A total of 36 SD rats were divided into 6 groups, each containing 6 rats. Repeated topical application of 1-cholro-2,4-dinitrobenzene(DNCB) was applied on the hair-removed abdomen of mice to induce AD [59]. The blank control group (I) was treated with acetone/olive oil (3:1), and the model group (II) was treated with DNCB in acetone/olive oil (3:1 *v/v*) [60]. The remaining experimental group with AD-induced rats (groups III, IV, V, and VI) were treated with hydrogel base (25 mg), marketed formulation (2% Candiderm cream, Glenmark) (25 mg), low dose (12.5 mg Tz) TTFsH, and high dose (25 mg Tz) TTFsH, respectively, on the dorsal skin of the back. Additionally, 5 mg of all formulations were spread on the ears of rats. All formulations were spread every day for four weeks in each group. After that, the erythema score and scratching scores were recorded for 30 min once a week using a digital camera [44,61].

#### 4.8.3. Histopathological Analysis

After the in vivo study of scratching and erythema scores and the skin irritation study, three rats from each group were anesthetized using slight ether (diethyl ether) and sacrificed at the end of the experiments [61,62]. A small portion of skin (1 cm^2^) tissue was removed and preserved in formalin (neutral buffered 10%). The formalin-fixed skin tissue samples were washed, dehydrated, and implanted in paraffin wax. The transverse section of embedded skin tissue (4µ) was cut by rotary microtome and gently heated to remove the paraffin wax. The sections were then hydrated with xylene, rinsed with ethanol, stained with hematoxylin-eosin, and examined using an optical microscope [42,62].

## 5. Conclusions

In this study, TFs were formulated and optimized to improve the penetration and localization of Tz in skin infected with AD. They were prepared using a simple lipid film hydration method and optimized using 3^2^ factorial designs using DOE version 13. From the result of the quadratic model, the B4 batch of TTFs was optimized to have a nanoscale vesicle size, narrow PDI, high EE%, and transdermal flux. Subsequently, it was dispersed into the hydrogel matrix containing different concentrations of Carbopol 980 and sodium CMC and evaluated for optimization. The in vitro drug release study showed that the F2 batch of TTFsH released 94.23 ± 0.984% Tz in up to 24 h, with the highest flux being 47.23 ± 0.82. The prepared low-dose TTFsH easily penetrated the stratum corneum and localized in the skin to treat AD. It also lowered the erythema score and the scratching score to zero, in comparison to the high dose TTFsH and the marketed formulation. The histopathological study also observed that a low dose of TTFsH potentially recovered the dermis, as well as the epidermis layer. Therefore, the present investigation concluded that a low dose of TTFsH is a very promising therapeutic molecule that offered high therapeutic efficacy on the skin for the effective suppression of a variety of atopic dermatitis symptoms.

## Figures and Tables

**Table 1:** Independent and dependent variables for the multilevel factorial design.

Independent Variables Concentration	Variable Levels		
	Low (0)	Medium (1)	High (2)
X_1_ = Phospholipon ^®^90H (mg)	50	100	150
X_2 =_ Sodium deoxycholate (mg)	15	30	45
Dependent Variables Goal			
Y_1_ = Particle size		Minimum in nanoscale	
Y_2_ = PDI		Minimum range from 0 to 1	
Y_3_ = Entrapment Efficiency		Maximum	

Legend of Tables in Appendix B.

**Table 2:** Different concentrations of phospholipon ^®^90H and sodium deoxycholate in TFs formulation using 3^2^ factorial design.

Formulation Code	X_1_ = Phospholipon ^®^90H (mg)	X_2_ = Sodium Deoxycholate (mg)
B1	100	15
B2	100	45
B3	50	30
B4	50	45
B5	100	30
B6	150	30
B7	150	15
B8	150	45
B9	50	15

**Table 3:** Summary of results of regression analysis for responses Y_1_, Y_2,_, and Y_3_.

Model	R^2^ Value	Adjusted R^2^	Predicted R^2^	F-Value	S.D.	CV%
Y_1_	0.96	0.94	0.72	21.09	7.68	4.05
Y_2_	0.94	0.92	0.84	26.03	0.78	0.83
Y_3_	0.92	0.96	0.89	23.07	1.12	1.93

**Table 4:** Characterization of prepared tioconazole transferosomes suspension (TTFs).

Formulation Code	Vesicle Size (nm) (Y_1_)	Flux (µg/cm^2^/h) (Y_2_)	%EE (Y_3_)	Zeta Potential (mV)	Polydispersity Index Y_2_ (PDI)
B1	130.42 ± 10.07	32.61 ± 0.12	72.55 ± 1.47	−19.46 ± 1.09	0.54 ± 0.09
B2	331.43 ± 14.41	34.92 ± 0.62	44.47 ± 2.90	−26.14 ± 2.04	1.03 ± 0.12
B3	223.35 ± 19.27	43.04 ± 0.34	88.12 ± 3.05	−18.84 ± 1.34	1.06 ± 0.22
B4	171.41 ± 9.03	48.23 ± 0.42	93.89 ± 2.41	−20.73 ± 1.23	0.36 ± 0.02
B5	263.85 ± 12.14	42.72 ± 0.18	44.86 ± 1.97	−14.02 ± 1.14	0.45 ± 0.04
B6	109.42 ± 9.07	35.51 ± 0.31	64.11 ± 1.54	−0.14 ± 0.12	0.32 ± 0.02
B7	169.82 ± 10.18	33.72 ± 0.41	80.89 ± 2.54	−0.87 ± 0.17	1.02 ± 0.14
B8	272.92 ± 9.47	32.93 ± 0.81	53.05 ± 1.43	−23.44 ± 1.47	0.19 ± 0.02
B9	207.62 ± 11.02	31.12 ± 0.11	88.12 ± 1.34	−19.34 ± 1.27	1.02 ± 0.03

All values are represented as mean ± SD (n = 3).

**Table 5:** Formulation of tioconazole-loaded transferosomes suspension.

Ingredients	Quantity
Drug (Tioconazole)	500 mg
Cholesterol	10 mg
Phospholipon^®^90H	50 mg
Tween 80	1 mL
Sodium Deoxycholate	45 mg
Ethanol	2 mL
Chloroform	1 mL
PBS (pH 7.4)	q.s.10 mL

**Table 6:** Physiochemical parameters of transferosome-loaded hydrogel ± SD.

Formulation Batches	Homogeneity	pH	Viscosity (Pa/s)	Spreadability (cm)	Flux (µg/cm^2^/h)	% Drug Content
F1	Poor	6.1 ± 0.16	3.6 ± 0.98	27.1 ± 0.12	41.08 ± 1.34	92.87 ± 1.47
F2	Excellent	6.5 ± 0.36	4.9 ± 0.36	26.6 ± 0.24	47.23 ± 0.82	95.72 ± 2.90
F3	Good	6.3 ± 0.16	5.8 ± 0.57	21.7 ± 0.33	44.63 ± 0.85	89.87 ± 1.66
F4	Good	7.1 ± 0.23	5.4 ± 0.46	27.6 ± 0.60	32.61 ± 1.02	90.73 ± 2.41
F5	Excellent	6.6 ± 0.17	6.4 ± 0.71	26.8 ± 0.24	30.42 ± 2.52	93.67 ± 1.90
F6	Good	6.6 ± 0.16	7.9 ± 0.56	22.6 ± 0.37	30.14 ± 0.95	89.45 ± 1.48

**Table 7:** The calculated correlation coefficients for the in vitro release of Tz from transferosomes employing different kinetic orders or systems.

Transferosomes Batches	Zero Order (R^2^)	First Order (R^2^)	Higuchi Model (R^2^)	Korsmeyer–Peppas Model (R^2^)
F1	0.8041	0.6927	0.9647	0.0981
F2	0.8172	0.6701	0.9678	0.0890
F3	0.8609	0.6781	0.9731	0.0822
F4	0.8371	0.6902	0.9726	0.0811
F5	0.8161	0.7012	0.9801	0.0922
F6	0.8091	0.7011	0.9756	0.0840

**Table 8:** Hydrogel base batches with different concentrations of gelling agent (Carbopol 980 or Sodium CMC).

Formulation Batches	Sodium MetaBisulphite (mg)	Sodium CMC (g)	Carbopol 980 (g)	Triethanolamine (in Drops)	Methyl Paraben (mg)	Propyl Paraben (mg)	Distilled Water (q.s) (mL)
F1	100	1	-	-	10	1	qs to 100
F2	100	1.5	-	-	10	1	qs to 100
F3	100	2	-	-	10	1	qs to 100
F4	100	-	1	2	10	1	qs to 100
F5	100	-	1.5	4	10	1	qs to 100
F6	100	-	2	6	10	1	qs to 100

## Data Availability

Not applicable.

## List of Abbreviations

TZ	Tioconazole
SCMC	Sodium carboxymethyl cellulose
TFs	Transferosomes
TTFs	Tioconazole transferosomes suspension
TTFsH	Tioconazole transferosomes suspension loaded hydrogel
AD	Atopic dermatitis
DOE	Design of experiments and statistical data analysis
PDI	Polydispersity index
EE	Entrapment efficiency
FT-IR	Fourier transform infrared
DSC	Differential scanning colorimetry
SEM	Scanning electron microscopy
TEM	Transmission electron microscopy
BCS	Biopharmaceutically classified system
CPCSFA	Committee for the Purpose of Control And Supervision of Experiments on Animals
DNCB	1-cholro-2,4-dinitrobenzene
SD	Sprague Dawley
IAEC	Institutional Animal Ethical Committee
SD	Standard deviation
R2	Regression coefficient

## Appendix A. Legend of Figures

**Figure Number**	**Description**
Figure 1a–c	Three-dimension surface response plot showing the effect of Phospholipon^®^90H concentration with respect to sodium deoxycholate concentration over the particle size, PDI, and % EE, respectively.
Figure 1d–f	Contour plot showing the effect of Phospholipon^®^90H concentration with respect to sodium deoxycholate concentration over the particle size, PDI, and % EE, respectively.
Figure 1g	Overlay plot of optimized area for batch B4 (in yellow).
Figure 2	Vesicle size and zeta potential of the optimum batch of TTFs. Batch B4 had a vesicle size of 171.4 nm ± 9.03 (a) and a zeta potential of −26.13 (b), which facilitated the cross of the stratum corneum and stability to the TFs vesicles. FE-SEM images of B4 optimized batch of Tz-loaded transferosomes suspension (TTFs) (c and d), which was spherical, well identified, unilamellar nanovesicles.
Figure 3	FTIR overlay of Tz, TTFs, and TTFsH indicated the coordination of Tz to phospholipon^®^90H and a sodium deoxycholate complex. (a) DSC thermogram of Tz and TTFs predicted the amorphous or partially amorphous state of Tz in TFs, which led to a high-energy state and high disorder, resulting in enhanced solubility (b).
Figure 4	In vitro permeation studies of Tz-loaded TFs in saline phosphate buffer, pH 6.5, using Franz diffusion cell with freshly shredded dorsal rat skin.
Figure 5	Cumulative % Tz release from different batches of TTFsH. The F2 batch showed the highest % Tz release and followed a Higuchi drug release mechanism. It showed an initial burst release followed by slow and sustained release of Tz.
Figure 6	Four weeks of treatment with TTFsH (low dose) resulted in significant recovery of the skin compared to the marketed formulation, as shown in decreased scratching score (a) and erythema score (b). Clinical features of SD rat skin after the topical treatment of hydrogel base, marketed formulation (Mkt. formulation), TTFsH (low dose), and TTFsH (high dose) on DNCB-induced AD (c).
Figure 7	Histopathology of (a) normal skin tissue, (b) model group (induced by DNCB), (c) tissue treated with hydrogel base, (d) tissue treated with marketed formulation, (e) tissue treated with TTFsH (low dose), and (f) tissue treated with TTFsH (high dose).

## Appendix B. Legend of Tables

**Table Number**	**Description**
Table 1	Independent and dependent variables for the multilevel factorial design.
Table 2	Different concentrations of phospholipon ^®^90H and sodium deoxycholate in TFs formulation using 3^2^ factorial design.
Table 3	Summary of results of regression analysis for responses Y_1_, Y_2,_, and Y_3_
Table 4	Characterization of prepared tioconazole transferosomes suspension (TTFs).
Table 5	Formulation of tioconazole-loaded transferosomes suspension (TTFs)
Table 6	Physiochemical parameters of transferosome-loaded hydrogel (TTFsH) with ± SD
Table 7	The calculated correlation coefficients for the in vitro release of Tz from transferosomes employing different kinetic orders or systems.
Table 8	Hydrogel base batches with different concentrations of gelling agent (Carbopol 980 or sodium CMC)
